# Coronary Artery Bypass Grafting in Patients with Advanced Left Ventricular Dysfunction: Excellent Early Outcome with Improved Ejection Fraction

**Published:** 2016-01-13

**Authors:** Mehrdad Salehi, Alireza Bakhshandeh, Mehrzad Rahmanian, Kianoosh Saberi, Mahdi Kahrom, Keivan Sobhanian

**Affiliations:** Imam Khomeini Hospital, Tehran University of Medical Sciences, Tehran, Iran.

**Keywords:** *Coronary artery bypass*, *Ventricular dysfunction, left*, *Myocardial revascularization*

## Abstract

**Background:** The prevalence of patients with severe left ventricular dysfunction (LVD) referred for coronary artery bypass grafting (CABG) is increasing. Preoperative LVD is an established risk factor for early and late mortality after revascularization. The aim of the present study was to assess the early outcome of patients with severe LVD undergoing CABG.

**Methods:** Between December 2012 and November 2014, 145 consecutive patients with severely impaired LV function (ejection fraction ≤ 30%) undergoing either on-pump or off-pump CABG were enrolled. The primary end point was all-cause mortality. Different variables (preoperative, intraoperative, and postoperative) were evaluated and compared.

**Results:** The mean age of the patients was 58.7 years (range, 34 to 87 years), and 82.8% of the patients were male. The mean preoperative LV ejection fraction was 25.33 ± 5.07% (10 to 30%), which improved to 26.67 ± 5.38% (10 to 40%) (p value < 0.001). An average of 3.85 coronary bypass grafts per patient was performed. Significant improvement in mitral regurgitation was also observed after CABG (p value < 0.001). Moreover, 120 patients underwent conventional CABG (82.8%) and 25 patients had off-pump CABG (17.2%). In-hospital mortality was 2.1% (3 patients). Patients who underwent off-pump CABG had higher operative mortality than did those undergoing conventional CABG despite a lower severity of coronary involvement and a significantly lower number of grafts (p value < 0.050). Conversely, morbidity was significantly higher in conventional CABG (p value < 0.050).

**Conclusion:** CABG in patients with severe LVD can be performed with low mortality. CABG can be considered a safe and effective therapy for patients with a low ejection fraction who have ischemic heart disease and predominance of tissue viability.

## Introduction

The prevalence of patients with severe left ventricular dysfunction (LVD) referred for coronary artery bypass grafting (CABG) is on the rise. Preoperative LVD is a well-known risk factor for early and late mortality after revascularization; and in these patients, bypass grafting remains a surgical challenge.^1^

Concerns over the revascularization of patients with severely impaired left ventricular (LV) function are poor prognosis, severe coronary artery disease, and suboptimal graft longevity because of poor distal runoff in patients with severe LVD and considerable myocardial scar burden. Accordingly, angina relief after revascularization might be deplorable. Thus, controversy still exists regarding the appropriate role of isolated CABG in patients with severe LVD.^2^

Recent advances in perioperative anesthesiology management, surgical procedures, and myocardial protection methods have conferred improved results; consequently, a greater number of patients with severe LVD are referred for CABG. Studies from different cohorts assessing the efficacy of surgical revascularization have demonstrated that these patients benefit the most from CABG, particularly if symptoms of angina — rather than heart failure — are evident.^3^ CABG in patients with severe myocardial dysfunction preserves the remaining viable myocardium, prevents additional myocardial deterioration, and induces the recovery of the systolic function of hibernated and ischemic myocardial segments. Nonetheless, the postoperative mortality rate in these patients ranges from 1.6 to 40.^4^

The aim of the present study was to evaluate the operative outcomes of patients with severe LVD who underwent isolated CABG (without aneurysmectomy, valve replacement, or other open heart procedures). The impact of preoperative and surgical variables on the early outcome was also analyzed.

## Methods

Between December 2012 and November 2014, 145 consecutive patients with severely impaired LV function (left ventricular ejection fraction [LVEF] ≤ 30%) undergoing either on-pump or off-pump CABG were enrolled.

The primary inclusion criteria for the patient selection were the diagnosis of coronary artery disease appropriate for CABG on preoperative angiography and LVEF ≤ 30% detected by both two-dimensional echocardiography and uni- or biplane contrast left ventriculography. 

Excluded patients from this series were comprised of those with end-stage renal failure on dialysis, valvular heart disease (including more-than-moderate mitral regurgitation), acute myocardial infarction, previous cardiac surgery, and need for ventricular aneurysmectomy or other surgical procedures.

Prospective cohort data were collected by trained list reviewers using standard data forms. The information elicited covered the patients' demographics; age; sex; risk factors such as diabetes, obesity, hypertension, smoking, addiction, hypercholesterolemia, renal failure, chronic obstructive lung disease, positive family history, peripheral vascular disease, carotid artery stenosis, cerebrovascular disease, prior myocardial infarction, and extent of coronary artery disease; and left main involvement. Additional data were obtained from the patients’ history and physical examination. Preoperative two-dimensional echocardiography and coronary angiography were performed in all the patients. 

The study protocol was approved by the institutional Medico-Ethical Review Committee, and written informed consent was signed by each patient prior to enrollment. 

Standardized anesthetic technique was used in all the patients during induction - including fentanyl, midazolam, and pancuronium - followed by the maintenance of isoflurane and propofol. For conventional CABG, after median sternotomy and harvesting of the left internal mammary artery and saphenous vein, cardiopulmonary bypass (CPB) was initiated after the administration of heparin at a dose of 300 to 400 IU/kg to achieve a target activated clotting time of 400 seconds or longer before the initiation of CPB. Cold St. Thomas blood cardioplegia was intermittently administered through the aortic root in all the patients and retrogradely through the coronary sinus (in 27 patients). CABG in the off- and on-pump patients was performed using conventional techniques, and complete revascularization was achieved in all the patients. On completion of all anastomoses and weaning from CPB (in the conventional CABG group), heparin reversal with protamine was performed to return the activating clotting time to normal levels.

The operative variables comprised priority of surgery (elective or urgent), technique used for coronary revascularization (on-pump or off-pump), number of bypass grafts, and CPB data.

At the end of surgery, the patients were transferred to the intensive care unit (ICU) and managed for ventilatory support, hemodynamic stabilization, temperature, fluid, and electrolyte balance. The patients were extubated as soon as they met the following criteria: consciousness with pain control, acceptable respiratory force and arterial blood gas, hemodynamic stability, normothermia, and no excessive bleeding. 

Postoperatively, our primary end point was all-cause mortality. Our other outcome variables consisted of low cardiac output state, reoperation for bleeding or tamponade, malignant arrhythmia, atrial fibrillation, postoperative myocardial infarction, blood transfusion, pleural effusion, sepsis or endocarditis, respiratory failure, transient ischemic attack or stroke, intra-aortic balloon pump, postoperative echocardiographic data, ICU and hospital length of stay, in-hospital death, and early mortality (≤ 30 days). 

All the data were collected prospectively on standard forms and entered into a computerized database. For the statistical analyses, the statistical software SPSS version 21 for Windows (SPSS Inc., Chicago, IL) was used. All the data were expressed as mean ± standard deviation. The clinical data were compared using the Mann-Whitney U test, Wilcoxon signed-rank test, chi-square test, and independent *t*-test when appropriate. A p value < 0.05 was considered statistically significant. 

## Results

Between December 2012 and November 2014, a total of 145 consecutive patients with EF of 30% or less and coronary artery disease amenable to surgical revascularization were assigned to either on-pump or off-pump CABG. The mean age of the patients was 58.7± 9.9 years (range, 34 to 87 years), and 82.8% of the patients were male. The number of patients who underwent conventional CABG was 120 (82.8%), and on-pump CABG was performed in 25 (17.2%) (Table 1). We performed a viability study to prove enough viable myocardium in all the patients preoperatively: cardiac magnetic resonance imaging in 23 patients, thallium perfusion scan in 65 patients, and stress echocardiography in the remainder. The average number of the preoperative risk factors in each patient was 1.66± 1.16, and diabetes was the most common one (37.9%) (Table 1). The mean preoperative LVEF was 25.33 ± 5.07% (10 to 30%), which improved to 26.67 ± 5.38% (10 to 40%) postoperatively (p value < 0.001). Significant improvement in the severity of mitral regurgitation was also observed after coronary revascularization (p value < 0.001). Nonetheless, there were no significant changes in the degree of aortic and tricuspid valve regurgitation severity after CABG on follow-up echocardiography.

The conventional CABG patients received 4.0 ± 0.80 grafts per patient, while the off-pump CABG patients had 3.12 ± 0.88 (p value < 0.001). The majority of the patients had three-vessel disease (80%), followed by two-vessel disease (18.6%) and single-vessel disease (1.4%). The severity of coronary involvement was significantly higher in the conventional CABG group than in the off-pump CABG group (p value < 0.001).

Intra-aortic balloon pump was used preoperatively in just 1 patient, and no other patients needed intra-aortic balloon pump support for low cardiac output state during the postoperative course. The mean number of platelet concentrates transfusions during hospitalization was 1.18 ± 0.865 units (range, 0 to 5), which was significantly lower in the off-pump CABG patients (p value < 0.001). The average ICU stay of the patients was 1.79 ± 1.74 days (range, 1 to 14), and 87% of the study population stayed for only 1 day. The mean hospital stay of the patients was 5.43 ± 2.12 days (range, 3 to 17), and the median length of stay was 4 days. ICU and hospital stay showed no significant differences between the conventional and off-pump CABG patients (p value = 0.582 and p value = 0.667, respectively). 

Additionally, 30-day mortality was 2.1% (2 patients in the off-pump group and 1 in the conventional group). Total mortality was lower in the conventional CABG group than in the off-pump CABG group (0.8% vs. 8.0%), and the difference was significant (p value = 0.022). Nonetheless, the overall morbidity was significantly higher in the conventional CABG patients (25.0% vs. 16.0%; p value = 0.031). A higher number of preoperative risk factors was correlated with a higher postoperative mortality rate (p value = 0.038) (Table 2). 

**Table 1:** Preoperative patients characteristics^*^

Age (y)	58.7±9.9 years
Gender	
Male	120 (82.8)
Female	25 (17.2)
Types of CABG	
On-pump	120 (82.8)
Off-pump	25 (17.2)
Number of involved vessels	
Single-vessel disease	2 (1.4)
Two-vessel disease	27 (18.6)
Three-vessel disease	116 (80.0)
Left main involvement	18 (12.4)
Intra-aortic balloon pump	1 (0.7)
NYHA class	
I	5 (3.4)
II	11 (7.6)
III	43 (29.6)
IV	86 (59.3)
Diabetes	55 (37.9)
Hypertension	51 (35.1)
Smoking	43 (29.6)
Myocardial infarction	36 (24.8)
Addiction	28 (19.3)
Hyperlipidemia	26 (17.9)
Chronic obstructive pulmonary disease	13 (8.9)
Renal failure	7 (4.8)
Cerebrovascular accident	5 (3.4)
Peripheral vascular disease	3 (2.1)

CABG, Coronary artery bypass grafting; NYHA, New York Heart Association

* Data are presented as mean±SD or n (%)

**Table 2:** Predictors of postoperative morbidity and mortality by univariate analysis

	Mortality (P value)	Morbidity (P value)
Age	0.208	0.237
Number of risk factors	0.125	0.038
Preoperative ejection fraction	0.960	0.891
Preoperative mitral regurgitation	0.928	0.307
Preoperative aortic insufficiency	0.589	0.983
Preoperative tricuspid regurgitation	0.098	0.150
Postoperative ejection fraction	0.477	0.772
Postoperative mitral regurgitation	0.718	0.830
Postoperative aortic insufficiency	0.564	0.909
Postoperative tricuspid regurgitation	0.088	0.173
Number of diseased vessels	0.384	0.124
Number of grafts used	0.750	0.454
Intensive care unit stay	0.001	< 0.001
Hospital stay	0.002	< 0.001
Off pump	0.022	< 0.001

## Discussion

Severe LVD has long been considered a contraindication for surgical coronary revascularization due to high operative risk and poor outcome. Patients with severe coronary artery disease and severely impaired LV function represent a high-risk group referred for CABG. High mortality and morbidity rates (2.7 to 33% and 30 to 67%, respectively) were reported even in recent series.^5^^-^^7^ Heart transplantation is also a valuable alternative option, but the number of heart transplantation procedures is limited by donor shortage such that only 10% of suitable patients finally undergo heart transplantation and many die on the waiting list. 

One of the persisting challenging areas of treatment for a cardiac surgeon is curing a patient with LVD. According to literature review, these patients have a better response to surgery with improved survival, functional status, and control of ischemic symptoms as well as decreased prevalence of sudden cardiac deaths caused by arrhythmias^8^^-^^10^ in comparison with medical therapies. It has also been revealed that medical therapies may lead to a poor outcome.^11^ Nonetheless, surgical coronary revascularization in severe LVD could result in high postoperative mortality due to perioperative low cardiac output syndrome. Recent advances in surgical procedures, improved myocardial protection, and perioperative anesthesiology care have conferred better outcomes; accordingly, a greater number of patients with severely impaired LV function undergo CABG. 

Conventional on-pump CABG is still the standard technique used in coronary artery surgery. Nevertheless, because of some probable adverse effects such as inflammatory response, use of cardioplegia, aortic cross-clamping, hypothermia, and multiorgan dysfunctions - off-pump CABG can also be an effective alternative to avoid such complications. In spite of its safety and efficiency over on-pump CABG, the technique has been criticized by many opponents regarding completeness of myocardial revascularization, graft patency, and long-term results. Of note, another advantage of off-pump CABG is regional ischemia in comparison with systemic ischemia of the body, which is performed in the on-pump technique. Additionally, some investigators have maintained that less intervention on the myocardium could lead to an ameliorated outcome. In our study, the selection of on- or off-pump surgery was made on a case-by-case basis, according to the patients' hemodynamic stability, extent of coronary artery disease, and health status as well as the surgeon’s preference.

Different cohorts have demonstrated that CABG with or without pump is not very different vis-à-vis morbidity and mortality.^12^ However, the enormous variety of factors such as coronary anatomy, myocardial preservation techniques, perioperative myocardial infarction, completeness of revascularization, renal dysfunction, redo surgery, and age may influence CABG outcomes. In the present research, we tried to diminish these interfering factors and as a result our sample encompassed patients with isolated CABG and not any other parallel operations such as valve surgery or LV aneurysm repair. 

In our study, data were collected from 145 consecutive patients with LVEF ≤ 30%, meeting the inclusion criteria. The baseline demographic information and preoperative variables are presented in Table 1.

The 2.1% hospital mortality rate reveals that even these patients with severely impaired LV function can undergo CABG relatively safely with good results. This operative mortality in our patient group is acceptable among other contemporary series.

In the postoperative follow-up, improvement in LVEF after surgical revascularization was duly demonstrated. The mean preoperative LVEF was 25.33 ± 5.07% (10 to 30%), which improved to 26.67 ± 5.38% (10 to 40%) postoperatively (p value < 0.001). 

A unifying impression behind our results is that surgical myocardial revascularization preserves already viable and functioning myocardial muscle against later infarction and recruits the hibernating myocardium. This recruitment leads to the objective improvement in LVEF and to the amelioration of congestive heart failure and functional class.^13^

Significant improvement in the severity of mitral regurgitation was also observed after coronary revascularization (p value < 0.001). This improvement in mitral regurgitation is explained by the restoration of myocardial function due to the provision of the blood flow to the previously dysfunctional regions of the ischemic or hibernating myocardium, which is the main cause of ischemic mitral regurgitation. Nonetheless, no significant changes in the degree of the severity of aortic and tricuspid valve regurgitation were observed after CABG on follow-up echocardiography. This is also expected due to the non-causative correspondence of the ischemic myocardium to aortic and tricuspid valve regurgitation. 

We believe that a number of perioperative and postoperative factors may have contributed to our good operative and short-term results such as the high percentage (all of patients) usage of internal mammary artery grafting and reasonable intraoperative and postoperative use of inotropic support. Additionally, complete myocardial revascularization and successful myocardial protection are important clues and predictors of favorable short- and long-term results after CABG in patients with poor LV function.

In conclusion, we showed that surgical revascularization conferred a low and acceptable short-term mortality rate and a potential benefit to LVEF and ischemic mitral regurgitation in patients with severely impaired LV function. 

The limitations to the present study were its short-term period of postoperative follow-up (30 days after operations) and its use of only echocardiographic LVEF indices for the evaluation of LV systolic function. In the univariate analysis of the entire group, the off-pump procedures were related as a significant risk factor for perioperative death.^14^ Even so, as the patients in our series were not randomized to either off- or on-pump CABG and had selection bias, this interpretation should be made with caution. Furthermore, this study was a prospective cohort study; the conclusions drawn should, hence, be pondered according to these constraints.

This study will be continued in order to run into more patients and assess changes and survival benefit in the later postoperative period.

## Conclusion

CABG in patients with severe LVD can be performed with low mortality. CABG can be considered a safe and effective therapy for low EF patients with ischemic heart disease and predominance of tissue viability.

## Notes:

***This paper should be cited as:*** Salehi M, Bakhshandeh A, Rahmanian M, Saberi K, Kahrom M, Sobhanian K. Coronary Artery Bypass Grafting in Patients with Advanced Left Ventricular Dysfunction: Excellent Early Outcome with Improved Ejection Fraction. J Teh Univ Heart Ctr 2016;11(1):6-10.
